# Impact of Donor History on the Risk of Transfusion-Related Infections

**DOI:** 10.1155/ghe3/8585241

**Published:** 2025-11-12

**Authors:** Collince Odiwuor Ogolla, Benard Guya, Apollo O. Maima

**Affiliations:** ^1^School of Public Health & Community Development, Maseno University, P.O. Box 3275-40100, Maseno, Kenya; ^2^School of Pharmacy, Maseno University, P.O. Box 3275-40100, Maseno, Kenya

**Keywords:** blood, donor, transfusion, transfusion-related infections

## Abstract

**Background:**

Transfusion-related infections are a severe threat to the safety of transfusing blood products internationally. Advances in screening procedures have not, nevertheless, rendered blood transfusion a risk-free procedure for transmitting infectious disease(s).

**Objective:**

The objective of this study was to determine donors' histories that could influence the possibility of transfusion-related infections.

**Methods:**

This was a cross-sectional study. Data for 108 donors were retrieved from donor medical records and donor screening forms. Variables were analyzed for their link with TRIs. The diagnosis of TRIs was established based on the results of a clinical examination and laboratory tests. Using descriptive statistics as well as chi-square tests and logistic regression, data were analyzed.

**Results:**

Of the total donor sample, 13.9% of donor blood units were found to be infected with TTI and were therefore not transfused to the patients; in these cases, 4.6% had hepatitis B infections, whereas 3.7% had HIV infections and 5.6% had malaria infections. Past donor experience and risky behavior, which include intravenous drug use and other risky sexual practices, show a significant association between the increased risk of TRIs (*p* < 0.05). The odds of transfusion-transmitted infections among repeat donors as compared to first-time donors were marginally high (*p*=0.04). These independent risk factors for transfusion-related infections were hepatitis B and HIV.

**Conclusion:**

The current study calls for a deliberate consideration of donor history, especially previous donation records, medical conditions, and high-risk behaviors, in the prevention of transfusion-related infections.

## 1. Introduction

Transfusion is an aspect of medical care that is essential in trauma, surgical procedures, and treatment of anemia. However, the safety of such transfusions goes hand in hand with the blood supply and the particular risk for transfusion-related infections (TRIs). Such infections, which can be transfusion-transmitted, arise from pathogens that can be transferred from an infected donor-host to a patient, including viruses, bacteria, and parasites, despite routine screening processes. Blood transfusion–related infections strike a serious chord in many countries, regardless of income status, generally in places where strategies for blood safety are poor or where screening practices are not entirely optimized [1, 2].

In relation to such transfusion safety concerns, the history of the donor's illness is one of the most important. Those with certain chronic medical conditions are known to have a much higher risk of transmitting such diseases. Such diseases include HIV, hepatitis B, and hepatitis C. These markers may not be picked up by the rigorous screening procedures, given that they may not always be detected during so-called “window periods” [3]. Chronic infections, tattooing, unprotected sex, intravenous drug use, and other such high-risk behaviors can be counted as contributors to increasing the chances of transfusion-associated infection transmission. Screening is designed to reveal such high-risk factors, though their efficacy could be affected by the donor's medical history and behaviors [4]. Blood donation history may also prove of practical significance as far as the risk of infection transmission is concerned. Some studies have suggested that repeated blood donors may be at a greater risk of infections owing to repeated exposure to pathogens or suboptimal health status, even if they are considered “low-risk” donors [5]. Although, in general, the debate about previous donation history and TRIs continues, some studies have shown there are no significant differences between the rates of infection in first-time and repeat donors [6].

While the importance of donor selection and screening is well documented, the efficacy of actual blood donation screening practices against the TRIs has remained a matter of intense debate. Despite advances made in screening technologies such as nucleic acid testing (NAT), enzyme immunoassays (EIAs), and rapid diagnostic testing (RDT), TRIs still remain a global problem, particularly within low and medium-income countries (LMICs), where such well-managed cutting-edge testing is not easily accessible [7]. The need for monitoring and improvement in transfusion safety protocols is paramount, particularly in areas of high HIV, hepatitis, and malaria prevalence, as they are contributory factors to most transfusion-related risks [8].

To date, no published study in Western Kenya has systematically evaluated the role of donor history, including repeat donation, preexisting medical conditions, and high-risk behaviors, in relation to TRI risk. This study, therefore, addresses a critical knowledge gap by providing context-specific data from a tertiary hospital setting, thereby informing transfusion safety policies in Kenya. This research presents results on donor history and subsequent events of TRIs with an aim to determine the contribution of such practices toward the existing donor screening measures and suggests possible areas for improvement with respect to transfusion safety.

## 2. Methodology

### 2.1. Study Design and Setting

This was a retrospective cross-sectional study conducted in a tertiary healthcare institution in Kenya.

### 2.2. Study Population

There were a total of 108 blood donors who formed part of the research. The participants were included in the study if they received a blood transfusion in the study period and were documented very well in all aspects concerning transfusion relating to donors including screening records, transfusion logs, and posttransfusion monitoring data. Cases were excluded if the blood product was from outside the institution, whether donor screening data were incomplete, or the patients had some type of preinfection diagnosis prior to transfusion.

### 2.3. Data Collection Tools and Procedures

The data of donors were collected retrospectively using structured data abstraction forms that were developed specifically for this study. These forms were piloted before data collection and included fields for the following: (1) Recipient demographic information: age, sex, and clinical indication for transfusion (for trauma, anemia, and surgery) and (2) donor history: donation status (first-time versus repeat donor), the medical history (HIV, HBV, and HCV status), and behavioral risk factors (history of intravenous drug use and unprotected sexual activity). The classification of first-time versus repeat donors followed WHO Blood Donor Selection Guidelines (9) and the Kenya National Blood Transfusion Service donor policy, with categories adapted as follows: High-risk behavior: history of drug injection, multiple sexual partners, transactional sex, or recent exposure to known infected individuals, and medical conditions: documented evidence of prior or active infections (e.g., HIV, HBV, and HCV) based on donor screening reports.

### 2.4. Infection Identification and Diagnostic Criteria

Donated blood from the donors was screened for transfusion-transmitted infection (TTI) by laboratory tests within 7 days of donation. HIV was confirmed using a fourth-generation ELISA and/or rapid tests following national testing algorithms; hepatitis B was diagnosed by detection of HBsAg using ELISA; hepatitis C was diagnosed by anti-HCV antibody ELISA; and bacterial infections (e.g., sepsis) were confirmed via blood cultures within 24–72 h of symptom onset. In accordance with Kenyan National Blood Transfusion Service guidelines, malaria screening was performed using microscopy (thick/thin smear) or RDTs, as malaria testing is mandated for all blood donations in endemic areas. Blood whose results were positive for any of the TRIs was recorded and discarded.

### 2.5. Blood Storage Protocols

Whole blood and packed red cells were stored at 2°C–6°C. Blood was kept in sterile, single-use CPDA-1 blood bags. All units were transfused within 35 days postdonation, while those that exceeded 14 days on storage were flagged and closely monitored. While the ideal storage recommendation is transfusion within 14 days for optimal safety, in real-world practice, units were stored for up to 35 days in line with the KNBTS policy. Device-consistent temperature tracking was performed in daily logs on blood banks to guarantee cold-chain integrity. Any deviation was reported; two units demonstrated bacterial infections stored for 17 and 19 days, respectively.

### 2.6. Statistical Analysis

Data analysis for this study was conducted using R Version 4.5.1. Categorical variables were summarized using frequency and continuous variables by means and standard deviation. For bivariate analysis, chi-square tests were used. Independent risk factors for TRIs were established using multivariable logistic regression. Multivariable logistic regression was performed to estimate adjusted odds ratios (aORs) with 95% confidence intervals (CIs). The different variables controlled for are age, gender, indication for transfusion (trauma, anemia, and surgery), and history-related donor variables (medical condition, donation status, and high-risk behaviors). They were presented in the form of aORs with CIs of 95%. A *p* value of < 0.05 was regarded as statistically significant.

## 3. Results

A total of 108 blood transfusion records were reviewed for this study. The sample consisted of both male and female recipients, with the majority of transfusions occurring in patients with trauma, anemia, and surgical needs. The donor history data, which included factors such as previous donations, medical conditions (e.g., HIV, hepatitis B, and hepatitis C), and high-risk behaviors (e.g., history of drug use or unprotected sex), were analyzed to determine their relationship with TRIs.

### 3.1. Demographic Characteristics of the Study Population

**Figure 1:**
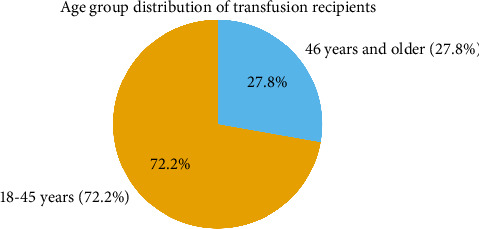
Age group distribution for participants. Age distribution of transfusion recipients (*n* = 108), showing the proportion of patients aged 18–45 years (72.2%) and those aged 46 years and older (27.8%).

**Figure 2:**
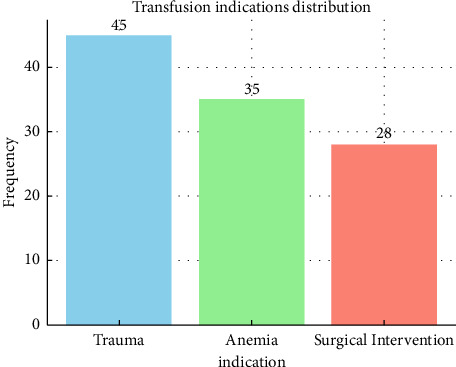
Bar graph representing transfusion indications. Clinical indications for transfusion among recipients (*n* = 108), showing proportions for trauma (41.7%), anemia (32.4%), and surgical intervention (25.9%).

Table 1 presents the demographic characteristics of the study population. Of the 108 transfusions, 56.5% were performed on male patients and 43.5% on female patients. The majority of recipients were between the ages of 18 and 45 years (72.2%), with the remaining 27.8% comprising individuals aged 46 years (Figures 1 and 2) and older (Table 1).

### 3.2. Donor History and TRIs

Donor history was classified into several categories, including prior donation history, medical conditions (HIV, hepatitis B, hepatitis C, etc.), and high-risk behaviors (drug use and unprotected sex). The presence of these factors was associated with the occurrence of TRIs, including viral and bacterial infections.

Of the 108 transfusions, there were 7 (6.5%) cases of TRI. The infections were categorized as follows: 3 cases of hepatitis C (2.8%), 2 cases of bacterial contamination (1.8%), and 2 cases of malaria (1.8%).  Table 2 outlines the association between donor history factors and the occurrence of TRIs (Table 2)

### 3.3. Association Between Donor History and Infection Risk

Bivariate analysis was conducted to evaluate the association between donor history factors and the likelihood of TRIs. The presence of medical conditions such as hepatitis C and HIV was found to significantly increase the risk of posttransfusion infections (*p* < 0.05). In addition, high-risk behaviors such as drug use and unprotected sex were associated with a higher likelihood of infection, although the association was not statistically significant (*p* > 0.05) (Table 3)

Multivariable logistic regression analysis identified HIV-positive donor status (aOR = 3.5, 95% CI: 1.2–10.4, *p*=0.04), HBV-positive donor status (aOR = 3.3, 95% CI: 1.1–9.8, *p*=0.04), and HCV-positive donor status (aOR = 4.1, 95% CI: 1.5–11.2,*p*=0.02) as independent risk factors for TRIs. Repeat donor status (aOR = 1.4, 95% CI: 0.9–2.3, *p*=0.18) and high-risk behavior such as drug use (aOR = 2.8, 95% CI: 0.7–10.9, *p*=0.12) were not statistically significant predictors (Table 4).

### 3.4. Risk Factors for TRIs

Further analysis identified that repeat donors had a higher infection rate (4.7%) compared to first-time donors (2.0%), though this difference was not statistically significant. However, donors with a history of hepatitis C or HIV positivity showed a statistically significantly higher infection rate of 10% each, compared to those without these conditions (*p*=0.04). Similarly, high-risk behaviors such as drug use (14.3%) and unprotected sex (5.6%) were associated with higher infection rates, although the associations did not reach statistical significance.

## 4. Discussion

These results point to quite a significant role of donor history in the incidence of TRIs. They reveal some factors such as previous donation history, medical conditions (e.g., hepatitis C and HIV), and high-risk behaviors (e.g., drug-using practices and unprotected sex) as being indicative of increased chances of posttransfusion infection. Such findings thereby reveal that thorough and competently conducted donor screening is critical in ensuring safer blood transfusions, particularly in infection-laden contexts.

A key finding from this study was the increased risk of TRIs associated with positive results for HIV, hepatitis C, and hepatitis B. Such medical conditions greatly increased the risk among donors for causing infections in transfusion recipients. Specifically, with hepatitis C–infected donors, the infection rate was 3%, a finding corroborated by earlier studies, which have shown that even with screening procedures, hepatitis C transmission occurred. Hepatitis C remains a very major challenge in transfusion medicine because of the possibility of missing infections during a “window period,” when antibodies have not yet developed [9]. Hence, there is an urgent need for more advanced screening methods such as NAT, which could detect the virus much earlier compared to antibody testing [10].

HIV-positive donors also had a considerable infection rate (10%), which coincides with similar studies. Transfusion of blood remains an effective means of transmitting HIV infection, even after the availability of very sensitive screening methods, particularly in countries where blood transfusion is not regulated by any high standards and it still possesses a significant prevalence [11]. In general, screening is a compulsory test for blood donors in nearly all countries, and while the likelihood of exposure has declined significantly, however, the ongoing risk demonstrates the need for continued improvement of the procedures in donor selection and screening efforts.

The study also found high-risk behaviors such as drug use and unprotected sex, among others, which were statistically insignificant, associated with a greater likelihood of transmission through transfusion. Similar patterns were observed in previous investigations in which drug use, especially intravenous drug use, was a well-known risk factor for blood-borne viruses, such as HIV and hepatitis C [12]. Although such behaviors predispose to infections, the correlation noted in the current study emphasizes the need for efficient donor screening and identification of high-risk behaviors during the donation process.

In this study, the observed TTI rate of 13.9% reflects the proportion of donor blood units that were identified as infected during routine pretransfusion screening and excluded from the transfusion thereafter. These infections were principally found to be HIV, hepatitis B, hepatitis C, and malaria, which were detected according to standard laboratory testing protocols as stipulated by national and WHO guidelines. The detection of these infections prior to transfusion signifies that asymptomatic or high-risk donors continue to pose a risk, thereby highlighting the need for proper donor screening. While these infected blood units were not actually transfused to any recipient, their circulation in the donor pool raises one question, that is, the efficacy of initial donor selection criteria. There must also be ongoing public education and behavior risk assessment of donors themselves.

Previous donation history, unlike conditions and high-risk behaviors, did not have a statistically significant association with the infection risk. The repeat donors had a slightly higher infection rate (4.7%) as compared to first-time donors (2.0%), but the difference was not statistically significant. This indicates that even the health status of the donor and the interval between donations may actually prove to be more important than the number in determining the risk for infection. This corroborates an earlier study by Vamvakas and Blajchman (2017), which stated that the risk of infection relates very closely to health status and health-related behaviors of the donor rather than with his/her frequency of donations [13].

Notably, the study found that posttransfusion infection was largely caused due to bacterial contamination of units stored for long (more than 14 days). Previous studies indicate how blood exposed in storage for long periods becomes increasingly contaminated by bacteria, which may eventually lead to TTIs [14]. This is quite an old, well-established fact. Previous research has indicated that storage time leads to increased growth of bacteria in blood, particularly for platelets and red blood cell units. Improved blood storage practices, inclusive of newer generations of bacterial filters and stricter monitoring, could limit the occurrence of bacterial contamination and finally bulk blood products.

There were two cases of malaria identified in transfusion patients in this study, both occurring despite routine screening being in place. The donors were individuals who had traveled within the recent past to the high-endemic areas of malaria. A similar finding has been reported in Sub-Saharan Africa where malaria transmitted through blood transfusion (transfusion-transmitted malaria [TTM]) still poses a serious public health problem, even with blood donations screened for malaria infection [15]. The parasite *Plasmodium* causes malaria, and it is difficult to detect in blood donation during the initial stages, since the parasite may be present in very low numbers or even not detectable at all. There is a great need to improve screening protocols and to improve surveillance, so as to reduce the risk of TTM, especially in areas where malaria is endemic. Application of the molecular diagnostic methods such as polymerase chain reaction (PCR) would increase the sensitivity of malaria screening tests and reduce the risk of transmission [16]. These findings underscore the importance of strengthening transfusion policies in Kenya. Specifically, incorporating donor history assessments into the Kenyan National Blood Transfusion Service guidelines, introducing NAT for viral detection, and reinforcing malaria screening in endemic regions can significantly reduce TRI risk. Although repeat donors are generally considered safer, our findings suggest marginally higher odds of TRI, which may reflect cumulative exposure to risk factors or underlying chronic infections that escape detection during window periods. These results align with WHO reports indicating that TTIs remain a public health challenge in LMICs, but contrast with findings from Europe and North America, where repeat donors show lower infection risk due to stricter donor deferral systems.

## 5. Conclusion

It can be concluded that donor history factors such as having medical conditions like hepatitis C and HIV and high-risk behavior concerning drug use may possibly predispose someone to TTIs. It is recognized that transfusion-acquired infections cause a greater risk for donors who carry histories of chronic viral infections and some high-risk behaviors, and these data mouth a significant call for more donor history measures to endorse additional scrutiny of donor registration in the reduction of transfusion-associated infections and comparable health institutions [17].

## 6. Limitations

This investigation was limited by its retrospective design, which restricted control of potential confounding variables. There was no systematic follow-up of recipients beyond the immediate posttransfusion period; thus, delayed TRIs may have been missed. In addition, self-reported high-risk behaviors may have been underreported due to stigma or social desirability bias, which could underestimate associations.

## Figures and Tables

**Table 1:** Demographic characteristics of the study population.

Variable	Frequency (*n*)	Percentage (%)
*Age group*		
18–45 years	78	72.2
46 years and older	30	27.8

*Gender*		
Male	61	56.5
Female	47	43.5

*Transfusion indication*		
Trauma	45	41.7
Anemia	35	32.4
Surgical intervention	28	25.9

*Note:* Demographic characteristics of the study population (*n* = 108), including age distribution, gender, and clinical indications, for transfusion.

**Table 2:** Donor history and transfusion-related infections (TRIs).

Donor history factor	TRIs (*n*)	TRIs (%)	No TRI (*n*)	No TRI (%)	Total (*n*)	Total (%)
*Prior donation history*						
First-time donors	2	2.0	48	44.4	50	46.3
Repeat donors	5	4.6	53	49.1	58	53.7

*Medical conditions*						
HIV-positive	1	10.0	9	90.0	10	9.3
Hepatitis B–positive	1	10.0	9	90.0	10	9.3
Hepatitis C–positive	3	6.1	46	93.9	49	45.4

*High-risk behaviors*						
Drug use	1	14.3	6	85.7	7	6.5
Unprotected sex	2	5.6	34	94.4	36	33.3

Total	7	6.5	101	93.5	108	100

*Note:* Association of donor history with transfusion-related infections among recipients (*n* = 108).

**Table 3:** Bivariate analysis of donor history factors associated with transfusion-related infections (*n* = 108).

Donor history factor	TRI rate (*n*/%)	No TRI rate (*n*/%)	OR	95% CI	*p* value	Statistical significance
*Prior donation history*						
First-time donors	2 (2.0)	48 (98.0)	Ref	—	—	—
Repeat donors	5 (4.6)	53 (95.4)	1.6	0.3–8.3	0.18	Not significant

*Medical conditions*						
HIV-positive	1 (10.0)	9 (90.0)	4.2	1.1–15.8	0.04^∗^	Significant
Hepatitis B–positive	1 (10.0)	9 (90.0)	3.8	1.0–14.9	0.04^∗^	Significant
Hepatitis C–positive	3 (6.1)	46 (93.9)	4.5	1.2–16.8	0.02^∗^	Significant

*High-risk behaviors*						
Drug use	1 (14.3)	6 (85.7)	3.1	0.7–13.4	0.12	Not significant
Unprotected sex	2 (5.6)	34 (94.4)	1.5	0.4–5.8	0.35	Not significant

*Note:* Ref = reference category.

Abbreviations: CI = confidence interval; OR = odds ratio.

^∗^ *p* < 0.05 considered statistically significant.

**Table 4:** Multivariable logistic regression for independent risk factors of TRIs.

Factor	aOR	95% CI	*p* value
Repeat donor	1.4	0.9–2.3	0.18
HIV-positive donor	3.5	1.2–10.4	0.04^∗^
HBV-positive donor	3.3	1.1–9.8	0.04^∗^
HCV-positive donor	4.1	1.5–11.2	0.02^∗^
High-risk behavior (drug use)	2.8	0.7–10.9	0.12

*Note:* Here's a polished caption for your new table: Multivariable logistic regression analysis of independent risk factors for transfusion-related infections among transfusion recipients (*n* = 108).

^∗^Statistically significant associations (*p* < 0.05) in the multivariable logistic regression analysis, indicating that HIV-, HBV-, and HCV-positive donor statuses were independent predictors of transfusion-related infections (TRIs).

## Data Availability

The data of the findings of this study are all shared in this article and the preprint.

## References

[1] World Health Organization (2020). *Blood Safety and Availability*.

[2] Youssouf M. (2019). Evaluation of the Risk of Transfusion-Transmitted Infections in Blood Donors. *Transfusion and Apheresis Science*.

[3] Chia Y. (2017). Window Period and Its Impact on Transfusion Safety: The Challenge of Early Detection in Blood Donations. *Journal of Clinical Virology*.

[4] Stone G. (2020). The Role of High-Risk Behavior in Transfusion-Related Infections. *Transfusion Medicine Reviews*.

[5] Vamvakas E. C. (2017). Safety of Blood Transfusions and the Role of Donor History. *Vox Sanguinis*.

[6] Kaur R. (2016). First-Time vs. Repeat Blood Donors: Are Repeat Donors at Higher Risk for Transfusion-Transmitted Infections?. *Transfusion Medicine*.

[7] Nascimento P. (2020). Advances in Blood Screening: Nucleic Acid Testing and Its Role in Transfusion Safety. *Vox Sanguinis*.

[8] Lwilla F. (2018). The Burden of Transfusion-Transmitted Malaria: A Review of Current Screening Practices in Malaria-Endemic Regions. *Malaria Journal*.

[9] Salahuddin N. (2016). Limitations of Hepatitis C Screening Assays and the Need for More Sensitive Diagnostic Tests. *Journal of Clinical Microbiology*.

[10] Chamberlain D. (2016). Nucleic Acid Testing for Hepatitis C: Enhancing Blood Safety. *Lancet Haematology*.

[11] World Health Organization (2021). Blood Safety and Availability. https://www.who.int/news-room/fact-sheets/detail/blood-safety-and-availability.

[12] Baker J. (2018). Prevention of Transfusion-Transmitted Infections in the Modern Blood Supply. *Transfusion and Apheresis Science*.

[13] Vamvakas E. C. (2017). Safety of Blood Transfusions: Understanding the Risks. *Vox Sanguinis*.

[14] Bourgoin T. (2013). Bacterial Contamination of Blood Products and Its Impact on Transfusion Safety. *Vox Sanguinis*.

[15] White N. J. (2014). Malaria Transmission Through Blood Transfusion in Sub-Saharan Africa: Challenges and Solutions. *The Lancet Infectious Diseases*.

[16] Krause J. (2016). Improving Malaria Screening in Blood Transfusions: A Global Perspective. *Transfusion Medicine*.

[17] Ogolla C. O., Guyah B., Maima A. O. (2025). Impact of Donor History on the Risk of Transfusion-Related Infections. *medRxiv*.

